# Global Metabolomic Profiling of Mice Brains following Experimental Infection with the Cyst-Forming *Toxoplasma gondii*

**DOI:** 10.1371/journal.pone.0139635

**Published:** 2015-10-02

**Authors:** Chun-Xue Zhou, Dong-Hui Zhou, Hany M. Elsheikha, Guang-Xue Liu, Xun Suo, Xing-Quan Zhu

**Affiliations:** 1 State Key Laboratory of Veterinary Etiological Biology, Key Laboratory of Veterinary Parasitology of Gansu Province, Lanzhou Veterinary Research Institute, Chinese Academy of Agricultural Sciences, Lanzhou, Gansu Province 730046, PR China; 2 National Animal Protozoa Laboratory and College of Veterinary Medicine, China Agricultural University, Beijing 100193, PR China; 3 Faculty of Medicine and Health Sciences, School of Veterinary Medicine and Science, University of Nottingham, Sutton Bonington Campus, Loughborough, LE12 5RD, United Kingdom; University of Wisconsin Medical School, UNITED STATES

## Abstract

The interplay between the Apicomplexan parasite *Toxoplasma gondii* and its host has been largely studied. However, molecular changes at the metabolic level in the host central nervous system and pathogenesis-associated metabolites during brain infection are largely unexplored. We used a global metabolomics strategy to identify differentially regulated metabolites and affected metabolic pathways in BALB/c mice during infection with *T*. *gondii* Pru strain at 7, 14 and 21 days post-infection (DPI). The non-targeted Liquid Chromatography-Mass Spectrometry (LC-MS) metabolomics analysis detected approximately 2,755 retention time-exact mass pairs, of which more than 60 had significantly differential profiles at different stages of infection. These include amino acids, organic acids, carbohydrates, fatty acids, and vitamins. The biological significance of these metabolites is discussed. Principal Component Analysis and Orthogonal Partial Least Square-Discriminant Analysis showed the metabolites’ profile to change over time with the most significant changes occurring at 14 DPI. Correlated metabolic pathway imbalances were observed in carbohydrate metabolism, lipid metabolism, energetic metabolism and fatty acid oxidation. Eight metabolites correlated with the physical recovery from infection-caused illness were identified. These findings indicate that global metabolomics adopted in this study is a sensitive approach for detecting metabolic alterations in *T*. *gondii*-infected mice and generated a comparative metabolic profile of brain tissue distinguishing infected from non-infected host.

## Introduction

The obligate intracellular Apicomplexan parasite *Toxoplasma gondii* is the etiological agent of toxoplasmosis, a common parasitic diseases that affects virtually all vertebrate species including humans [1]. Humans or animals become infected congenitally from an infected mother to the fetus, by ingestion of the parasite tissue cysts from undercooked or uncooked meat or by drinking water contaminated with oocysts shed in feline feces [2]. Usually, the clinical spectrum of the disease varies, depending on the host genetic background and genotype of the infecting strain, from asymptomatic infection in immune-competent people to severe illness in pregnant women and immunocompromised individuals [3]. Acute infection caused by the rapidly proliferating tachyzoites is efficiently controlled by the immune system [4]. Tachyzoites then transform into a slowly replicating stage (i.e. bradyzoites) enclosed in tissue cysts, and remain dormant within the central nervous system (CNS) and muscles [5]. In immuno-compromised individuals, latent infection can be reactivated and causes fatal consequences. For example, toxoplasmic encephalitis (TE) in AIDS patients is caused by the reactivation of quiescent infection in CNS when CD4^+^ cells become <200 per μl [6].

Although infection in immune-competent people is generally benign, accumulating evidence indicated that *T*. *gondii* infection could modify the host’s behavior and impair its learning capacity and memory [7]. Further evidence linked *T*. *gondii* infection to several mental disorders, such as schizophrenia, mood disorders and suicidal behavior [8]. The pathogenic mechanisms underlying *T*. *gondii*-induced behavioral and neuronal disorders remain largely unknown. During *T*. *gondii* infection, host undergoes many pathophysiological changes including metabolic, proteomic, histological changes, in order to maintain homeostasis and adapt to the conditions that is required for its survival under challenge caused by the infecting parasite. *T*. *gondii* can invade microglia, astrocytes, and neurons, with the majority of cysts are found in neurons [9,10]. Microglia and astrocytes play important roles in intracerebral pathogen control by the production of antiparasitic and immunomodulatory effectors [11,12]. But, the functional role of neurons is still largely unknown. In chronic infections, neuronal death and lesions are caused by the production of nitric oxide (NO) and inflammatory cytokines from immune cells [13]. Although much has been learned from such studies, they focus on individual molecules and failed to reveal the global effects of infection on the host. There is a need to elucidate the neuro-pathogenicity of this parasite and to identify infection-specific biomarkers that differentiate between acute and chronic toxoplasmosis.

In recent years, the effects of *T*. *gondii* infections on the host’s brain have been studied with comprehensive methods, such as transcriptomics and proteomics to identify differentially expressed genes and proteins [14,15]. Metabolic profiling (i.e. metabolomics) is an emerging field that involves identification and quantification of numerous low molecular weight compounds in various biological samples. This approach provides a powerful means for the measurement of the chemical phenotypes that are the net result of all activity on the transcriptome and proteome levels; therefore, it provides an integrated global profile of the status of test organism [16–18]. Previous studies have focused only on targeted metabolites, such as dopamine and homovanillic acid [19,20]. However, toxoplasmosis is very likely associated with a complex array of chemical reactions and metabolites that stem from a diverse set of metabolic pathways associated with infection and inflammation. Therefore, we hypothesized that due to changes in brain function and metabolism caused by *T*. *gondii* infection, a characteristic pattern of metabolites would also be detectable.

The aim of this study was to determine whether metabolic profiling could be a reliable tool to detect alteration in the metabolome of brain of *T*. *gondii*-infected mice. For this purpose, BALB/c mice were experimentally infected with *T*. *gondii* Pru strain, and a LC-MS spectroscopy-based metabolomics approach was used to identify a characteristic metabolic ‘biomarker pattern’ in the brain of infected mice over the course of infection. The ability to discover significantly altered individual metabolites or metabolic pathways linked to *T*. *gondii* infection has been achieved. Physical recovery from acute toxoplasmosis has been correlated with biomarkers, which can be used as predictors of disease progression during *T*. *gondii* infection.

## Material and Method

### Ethics statement

All animal procedures were approved by the Animal Ethics Committee of Lanzhou Veterinary Research Institute, Chinese Academy of Agricultural Sciences (Permit No. LVRIAEC2014-002). All experiments were performed in strict compliance with requirements of the Animal Ethics Procedures and Guidelines of the People's Republic of China. We monitored infected mice for adverse health issues and efforts were made to minimize suffering. All infected mice were humanely euthanized by cervical dislocation under isoflurane inhalation anesthesia.

### Mice

Female BALB/c mice were purchased from Lanzhou University, China. All mice had free access to food and water and were kept in a temperature-controlled room (22 ±0.5°C) on reverse 12/12 h light/dark cycle. Mice were acclimated for five to seven days before being used in the experiment. Prior to initiation of the work mice were weighed (weight>20g) to establish a baseline for detecting reduction in body weight caused by infection.

### The parasite strain

*Toxoplasma gondii* type II Prugniuad (Pru) strain was maintained throughout the experiment via oral inoculation of cysts in mice. Cysts used in this study were isolated from brain tissues of infected BALB/c mice 40 days post-infection (DPI). Following anesthetizing animals, the brains of the infected mice were removed and homogenized in a tissue homogenizer under sterile conditions. Then, the number of cysts was counted and diluted to 100 cysts/ml in phosphate buffered saline solution (PBS).

### Experimental infection and parasite detection in mice

Six- to eight-week-old BALB/c mice (*n* = 6 per group) were either left uninfected or inoculated orally with freshly prepared 10 cysts of *T*. *gondii* Pru parasites using a small gavage needle. Body weight was measured daily for 21 DPI, and all mice were checked daily for the development of clinical signs characteristics of toxoplasmosis. Six mice from each of infected and control groups were sacrificed at 7, 14 and 21 DPI. Samples from blood, liver, spleen, lung, small intestine and kidney were collected for the confirmation of the presence of *T*. *gondii* as previously described [21]. Briefly, genomic DNA was extracted from representative body tissue samples using TIANamp Genomic DNA kit (TianGen™, Beijing, China) according to the manufacturer’s instructions. Specific PCR that targets 35-fold repeated B1 gene was applied to detect *T*. *gondii* infection in mice tissues using the specific primers (5′-AAC GGG CGA GTA GCA CCT GAG GAG–3′ and 5′-TGG GTC TAC GTC GAT GGC ATG ACA AC–3′). Positive control of DNA from parasites and negative controls (PBS) were included in each test. Also, pairs of adjacent 5-mm square sections from brain samples collected at different time points post infection were fixed in 10% neutral buffered formalin solution for 2 weeks, dehydrated in a graded series of ethanol, embedded in paraffin wax, and then cut into 5μm-thick serial sections on a microtome. They were then stained with hematoxylin and eosin [22]. Pathological changes were observed under optical microscope.

### LC-MS-based metabolomics

Unbiased metabolomic profiles of brain samples were obtained by using High Performance Liquid Chromatography-Mass Spectroscopy (HPLC-MS). To minimize any potential changes to the tissue metabolome, mice forebrains were rapidly removed and flash-frozen in liquid N2 and stored at −80°C until processing. Nearly 100mg sample was homogenized in 500 μl of a solvent of acetonitrile-water (7:3), treated with ultrasonic wave for 15 min. The mixture was centrifuged at 12,000rpm for 15min, at 4°C. Supernatant (200 μl) was transferred to a new 1.5 ml polypropylene tube before liquid chromatography separation.

The LC-MS system was run in a binary gradient solvent mode consisting of 0.1% (v/v) formic acid/water (solvent A) and 0.1% (v/v) formic/methanol (solvent B). The flow rate was 0.2 mL/min. A C18 column (Agilent Technologies, Santa, Clara, CA) (1.8 μm, 100×2.1 mm) was used for all subsequent analysis. The linear gradient was as follows: 5% B at 0 min, 5% B at 2 min, 95% B at 17 min, and 95% B at 19 min.

Sample analysis was carried out in both positive electrospray ionization (ESI+) and negative ion (ESI−) modes. The column temperature was 45°C and the injection volume was 5 μl. LC-MS data was acquired using an Agilent 1290 Infinity LC System coupled to an Agilent 6530 Accurate-Mass Quadrupole Time-of-Flight (Q-TOF) (LC-Q/TOF-MS). The mass scan range was 100–1000 m/z. For positive ion mode, the capillary and cone voltage were set at 4 kV and 35 kV, respectively. Nitrogen was used as the dry gas. The desolvation gas was set to 600 L/h at 350°C; the cone gas was set to 50 L/h and the source temperature was set to 100°C. For negative ion mode, the capillary and cone voltage were set at 3.5kV and 50 kV, respectively. The desolvation gas was set to 700 L/h at 300°C; the cone gas was set to 50 L/h and the source temperature was set to 100°C. The mass spectrometry was operated in V optics mode and the data acquisition rate was set to 0.03s, with interscan delay of 0.02s. To ensure accuracy and reproducibility LockSpray was used in all analyses and Leucine-enkephalin was selected as the lockmass.

### LC-MS data processing and statistics

Agilent MassHunter Workstation QTOF Acquistition software (B.03.01) was used for data acquisition. LC-MS raw data files were converted into common format (.mzdata), which was followed by data processing through the XCMS toolbox (http://metlin.scripps.edu/xcms/) [23]. Peak picking, peak grouping and retention time correction were all involved in data pre-treatment, which were done through the XCMS software that was implemented with the freely available R statistical language (v 2.13.1).

The retention time (RT)-m/z data pairs were used to indentify ion intensities of those detected peaks. The consistent variables (ion intensity information) were obtained by filtering peaks with 80% missing values. In order to remove the offsets and adjust the importance of high and low abundance metabolites to an equal level, the data was preprocessed by both mean-centering and variance-scaling prior to multivariate analysis. Then, the resulting three-dimensional matrix that includes assigned peak indices (retention time-m/z pairs), sample names and variables further went through the multivariate data analysis.

The resulting scaled datasets were imported into SIMCA-P+ 11.0 (Umetrics, Umea, Sweden) for processing using principal components analysis (PCA), PLS-discriminant analysis (PLS-DA) and orthogonal partial least-squares-discriminant analysis (OPLS-DA), which were carried out to visualize the maximal metabolic alterations between each group at all time points. Metabolites found to be highly similar or discriminatory were compared to investigate differential alteration at various time-points post infection. The differential metabolites were selected when the statistically significant threshold of variable influence on projection (VIP) values obtained from the OPLS-DA model was larger than 1.0. Meanwhile, the *p* values from a two-tailed Student’s *t*-test on the normalized peak areas were less than 0.05 [24]. Log2 fold change (FC) based on metabolite abundance was used to show how these selected differential metabolites varied between groups. The positive value indicates the level of a specific metabolite is increased. Meanwhile, the LC-MS abundance data set was imported into MeV software for heatmap generation. Diagnostic model was constructed by the marker metabolites that were extracted from the combining VIP value, *p* value and fold change. The classification performance (specificity and sensitivity) was assessed by the area under the curve (AUC) of the receiver-operator characteristic (ROC) curves using SPSS software.

### Metabolite Identification

Putative metabolites were first derived by searching the exact molecular mass data from redundant m/z peaks against the online HMDB (http://www.hmdb.ca/), METLIN (http://metlin.scripps.edu/) and KEGG (www.genome.jp/kegg/) databases. A specific metabolite was sieved out when a match with a difference between observed and theoretical mass was less than 10 ppm. Then the metabolite molecular formula of matched metabolites was further identified by the isotopic distribution measurement.

## Results

### Parasite infections in mice

**Fig 1:**
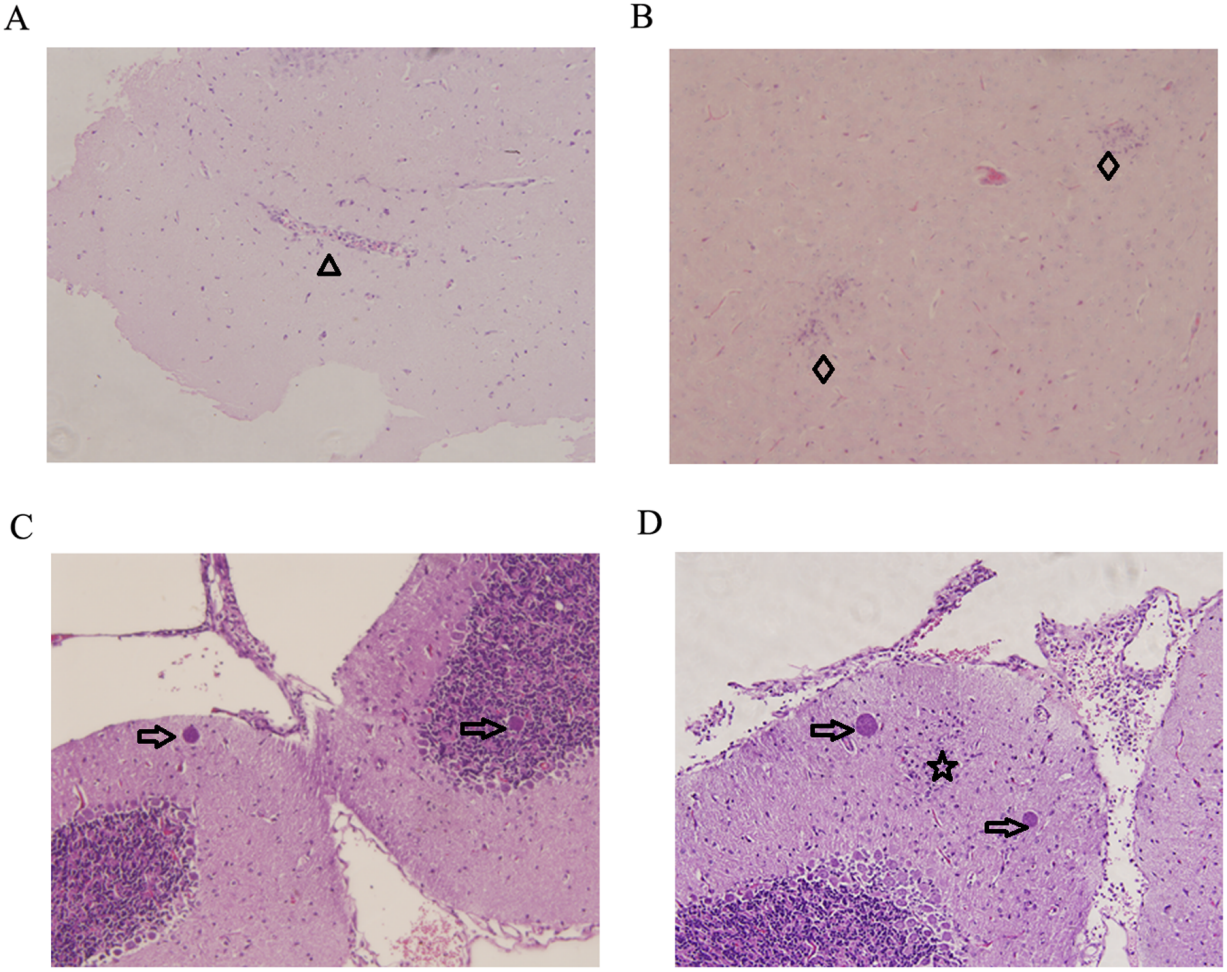
Histopathological lesions in mouse brain infected with *Toxoplasma gondii* Pru strain (10 cysts per mouse by oral route). (A)&(B) Infected mice brain collected on day 14 PI. (A) Perivascular cuffing (triangle). (B) Glial nodule (diamond). (C)&(D) Infected mice brain collected on day 21 PI. (C) Tissue cysts (arrow). (D) A glial focus (star) is found in the grey matter of the infected mice brain on day 21 PI. On both sides of it lie the tissue cysts (arrow). Magnification (200×).

Through the detection of *T*. *gondii* B1 gene, all infected mice were tested positive for *T*. *gondii* infection (data not shown). Mice infected by *T*. *gondii* Pru strain showed normal body weight, slight decline of feed consumption and slight signs of illness on day 7. However, physical condition of mice worsened over time and showed the typical clinical features of toxoplasmosis by 10 DPI, including anorexia, weight loss, edema and messy hair. More than half of the infected mice began to return to healthy status on day 14 post infection. Mice in the control group appeared normal and were tested negative for *T*. *gondii* infection during the whole experiment. Using hematoxylin and eosin (HE) staining, brain samples (7, 14 and 21 DPI) were examined for the severity and distribution of inflammation, tissue destruction, cell degeneration, and the numbers of cysts. Uninfected control mice did not show any histological changes. No significant pathologic changes were noted on 7 DPI. Mice in the infected group presented perivascular cuffing and glial nodule on 14 DPI, as shown in Fig 1A and 1B, respectively. More lesions occurred as infection progressed. On 21 DPI, the number of purkinje cells increased in the infected mice brains, which might be necessary for the survival of olivary neurons and cerebellar granule cells [25]. Tissue cysts were intact and found multifocally throughout many sections, which appeared in the proximity of glial foci, as shown in Fig 1D.

### Changes in brain metabolic profile with disease progression

**Fig 2:**
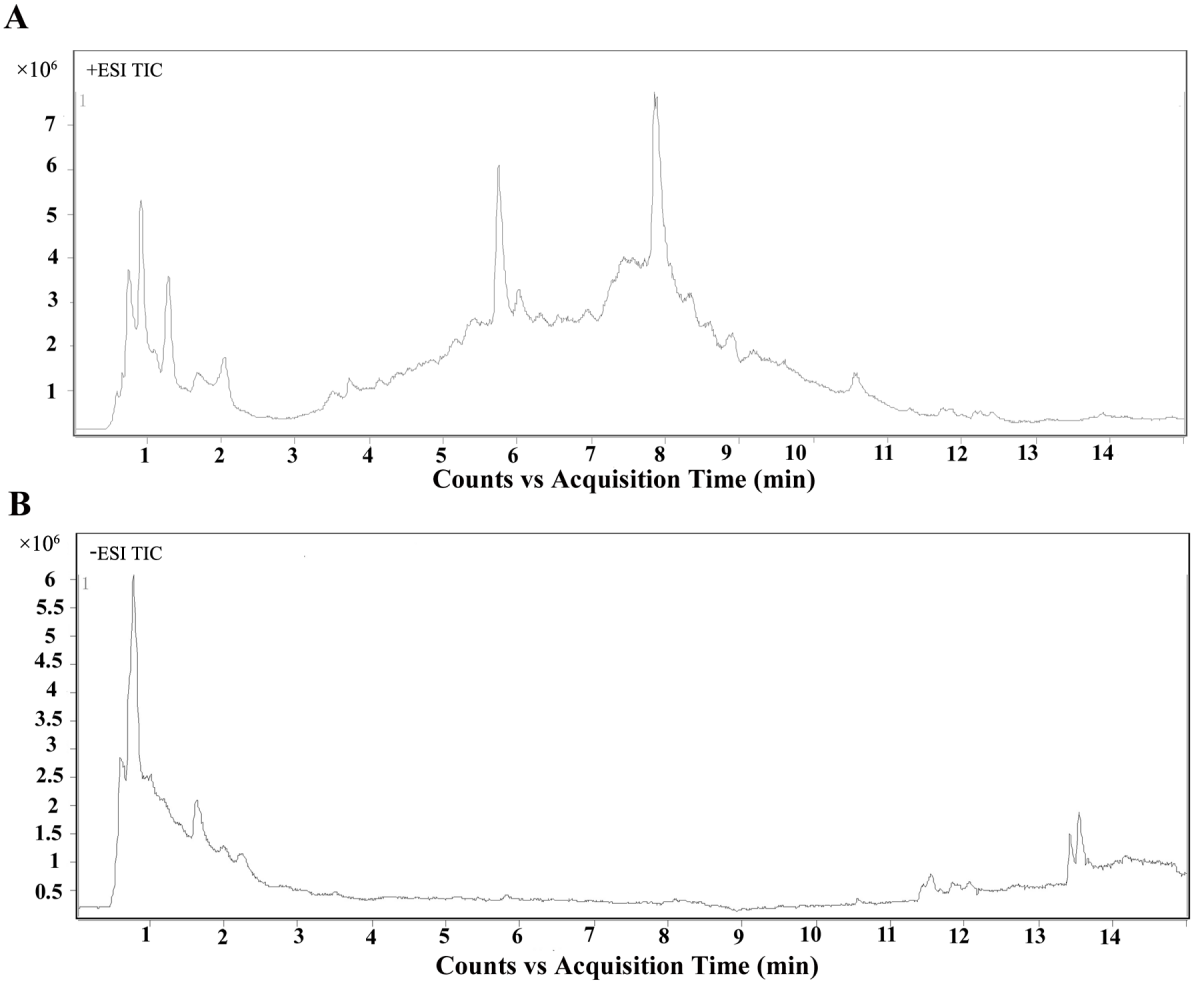
Metabolites changing significantly with infection. Typical TIC chromatograms (TIC) of mice brain obtained in the positive ion mode (ESI+) (A) and negative ion mode (ESI-) (B).

**Fig 3:**
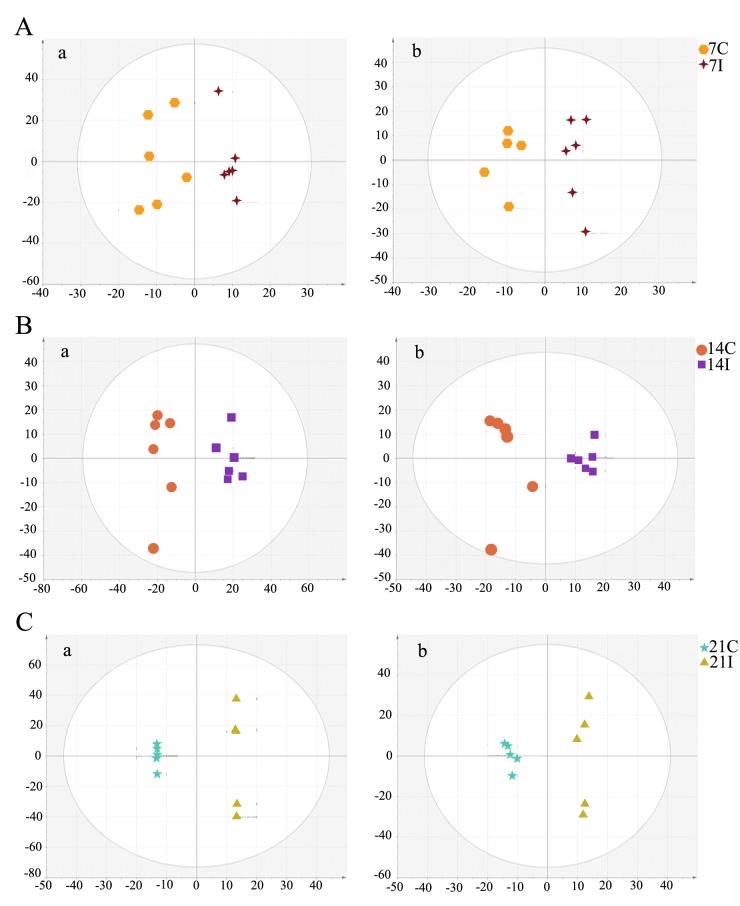
Orthogonal Partial Least Square Discriminant Analysis (OPLS-DA) score plot shows an excellent separation between infected mice groups and control groups. (A) The 7 day infected group (7I) and the 7 day control group (7C); (B) The 14 day infected group (14I) and the 14 day control group (14C); (C) The 21 day infected group (21I) and the 21 day control group (21C). In OPLS-DA score plot, each data point represents one mouse brain sample, and the distance between points in the plot indicates the similarity between samples. a. ESI+, b. ESI-. X axis is PC1, y axis is PC2.

Typical LC-MS base peak intensity (BPI) chromatogram of brain samples from different infection stages (7d, *n* = 6; 14d, *n* = 6; 21d, *n* = 5) and their corresponding control groups are shown in Fig 2. Using the LC−MS analysis protocol and subsequent processes, we detected approximately 2755 retention time-exact mass pairs, 1065 in negative ionization mode and 1690 in positive ionization mode. To analyze the profiling data from XCMS and identify samples that are serious outliers, PCA was performed. As shown in S1 Fig, the ESI+ model explained 68.9% of the variation in the metabolic profiling [R^2^X 68.9%] with a predictability of 45.3% [Q^2^ 45.3%]. The ESI- model explained 67.2% of the variation in the metabolic profiling [R^2^X 67.2%] with a predictability of 37.9% [Q^2^ 37.9%]. No outliers were observed in the data. Generally, the model is believed to be reliable when the R^2^X>0.4. The PCA results showed that the infected and control mice brain samples were not clearly separated. All subsequent analyses were carried out by Orthogonal Partial Least Square Discriminant Analysis (OPLS-DA). As shown in Fig 3, the OPLS-DA score plot showed distinct clustering of the infected mice groups and their corresponding control groups. Changes in metabolite profiles (positive or negative ion mode) over the time course of toxoplasmosis were similar, which indicated the metabolites detected in both positive and negative ion modes might evolve over the time course of the disease.

### Global metabolic variations between infected and control mice

**Fig 4:**
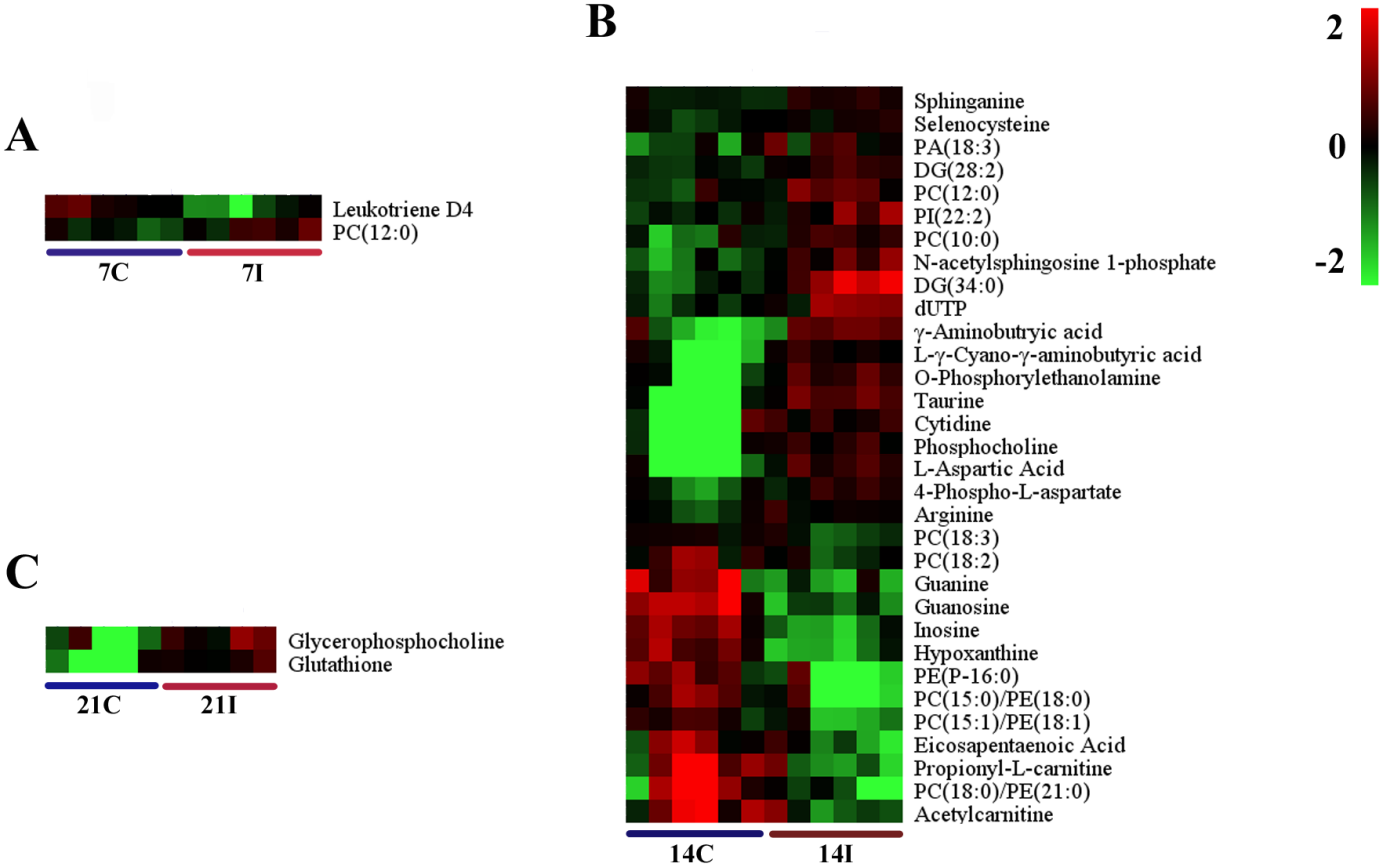
Comparison of the mice brain metabolomes during the course of infection. Heat maps representing the significantly changed metabolites between infected groups and the corresponding control groups in ESI+ mode (A, B and C). Normalized metabolite abundance (log2 transformed and row adjustment) are visualized as a color spectrum and the scale from least abundant to highest ranges is from -2.0 to 2.0. Green indicates low expression, whereas red indicates high expression of the detected metabolites. (A) 7D infected group (7I) vs 7D control (7C); (B) 14D infected group (14I) vs 14D control (14C); (C) 21D infected group(21I) vs 21D control (21C).

The differential metabolites associated with toxoplasmosis were identified according to VIP threshold (VIP >1) and the *P*-value of student’s *t*-test after FDR correction. These identified metabolites include amino acid, peptide, carbohydrate, lipid, nucleotide, cofactors and vitamins, and are involved in a wide range of biological pathways. The statistically significant metabolites detected at different time points, their m/z values, log2 fold changes (FC) and descriptions are shown in Table 1. Results showed that the number of significantly different metabolites varied during the infection process and only a small set of metabolites exhibited detectable periodic fluctuation nature. On day 7 (R^2^X = 0.277, R^2^Y = 0.889, Q^2^ = -0.837 ESI+; R^2^X = 0.302, R^2^Y = 0.927,Q^2^ = -0.346 ESI-) (Fig 3A), the levels of phosphatidylcholine (PC) (12:0), phosphatidylethanolamine (PE) (18:2), UDP-N-acetyl-D-galactosamine and aconitic acid were found to be higher in the infection group relative to healthy control group, whereas the levels of leukotriene D4 and inosine concentrations were found to be lower (Fig 4A, S2A Fig). Among these metabolites, leukotriene D4 is an important inflammatory mediator, which is formed through a cascade metabolism pathway from arachidonic acid [26].

**Table 1:** List of differentially-expressed metabolites identified at different stages of infection (infected “I” vs control “C”).

	VIP	m/z	*R*t (min)	metabolites	F.C.*	*p*-value	Metabolic pathways
**7I/7C(+)**	2.897	454.2596	5.79	PC(12:0)	0.58	2.56E-02	Not known
	3.370	497.2694	4.82	Leukotriene D4	-1.03	5.74E-03	Arachidonic acid metabolism
**7I/7C(-)**	2.275	476.2775	11.57	PE(18:2)	0.84	4.16E-02	Not known
	2.841	606.0742	1.68	UDP-N-acetyl-D-galactosamine	0.73	5.10E-03	Amino sugar and nucleotide sugar metabolism
	2.756	267.0733	0.85	Aconitic acid	0.33	7.65E-03	TCA cycle
	2.389	173.0088	1.09	Inosine	-0.25	2.98E-02	Purine metabolism
**14I/14C(+)**	1.363	524.3713	13.25	PC(18:0)/ PE(21:0)	2.11	2.72E-02	Not known
	1.809	284.0995	1.24	Guanosine	2.09	6.32E-04	Purine metabolism
	1.415	152.0565	1.24	Guanine	2.03	2.02E-02	Purine metabolism
	1.356	218.1388	0.95	Propionyl-L-carnitine	1.86	2.82E-02	Not known
	1.807	137.0457	1.26	Hypoxanthine	1.8	6.46E-04	Purine metabolism
	1.867	269.0888	1.26	Inosine	1.79	2.64E-04	Purine metabolism
	1.537	438.2982	12.43	PE (P–16:0)	1.54	9.16E-03	Not known
	1.493	482.3246	13.09	PC(15:0)/ PE(18:0)	1.52	1.25E-02	Not known
	1.257	204.1231	0.87	Acetylcarnitine	1.48	4.64E-02	Not known
	1.249	303.2323	12.57	Eicosapentaenoic Acid	1.2	4.82E-02	Biosynthesis of unsaturated fatty acids
	1.555	480.3089	12.39	PC(15:1)/ PE(18:1)	0.98	8.03E-03	Not known
	1.246	520.3401	11.76	PC(18:2)	0.79	4.88E-02	Not known
	1.393	518.3222	12.22	PC(18:3)	0.42	2.31E-02	Not known
	1.315	163.9779	0.74	Selenocysteine	-0.3	3.49E-02	Selenocompound metabolism
	1.385	302.3059	10.64	Sphinganine	-0.33	2.41E-02	Sphingolipid metabolism
	1.270	175.1189	0.72	Arginine	-0.37	4.37E-02	Arginine and proline metabolism
	1.814	509.4306	5.87	DG(28:2)	-0.6	5.90E-04	Not known
	1.396	214.0116	0.91	4-Phospho-L-aspartate	-0.62	2.27E-02	Glycine, serine and threonine metabolism
	1.428	454.2596	5.79	PC(12:0)	-0.73	1.87E-02	Not known
	1.469	426.2259	4.73	PC(10:0)	-0.74	1.45E-02	Not known
	1.299	433.233	4.70	PA(18:3)	-0.75	3.79E-02	Not known
	1.262	653.3687	7.47	PI(22:2)	-0.81	4.53E-02	Not known
	1.600	468.992	5.57	dUTP	-1.14	5.63E-03	Pyrimidine metabolism
	1.633	422.2641	7.59	N-acetylsphingosine 1-phosphate	-1.2	4.25E-03	Not known
	1.588	129.0659	1.37	L-γ-Cyano-γ-aminobutyric acid	-1.28	6.21E-03	Not known
	1.523	104.0714	1.37	γ-Aminobutyric acid	-1.38	1.01E-02	Not known
	1.397	244.0932	1.46	Cytidine	-1.47	2.26E-02	Pyrimidine metabolism
	1.677	142.0264	1.33	O-Phosphorylethanolamine	-1.47	2.86E-03	Glycerophospholipid and sphingolipid metabolism
	1.625	597.5586	7.76	DG(34:0)	-1.78	4.57E-03	Not known
	1.707	184.0735	1.37	Phosphocholine	-1.8	2.12E-03	Glycerophospholipid metabolism
	1.790	134.0449	1.38	L-Aspartic Acid	-1.91	8.10E-04	Alanine, aspartate and glutamate metabolism
	1.852	126.022	1.39	Taurine	-2	3.35E-04	Primary bile acid biosynthesis; Taurine and hypotaurine metabolism
**14I/14C(-)**	1.458	436.2827	12.10	PE(P–16:0/0:0)	1.23	2.85E-02	Not known
	1.711	327.2325	14.66	Docosahexaenoic acid	1.06	6.13E-03	Biosynthesis of unsaturated fatty acids
	1.522	452.2777	11.85	PC(13:0)/ PE(16:0)	1.01	2.04E-02	Not known
	1.387	480.3088	12.71	PC(15:0)/ PE(18:0)	1	4.00E-02	Not known
	1.453	528.3086	12.09	PE(22:4)	0.66	2.91E-02	Not known
	1.543	303.2326	14.83	Arachidonic Acid	0.6	1.82E-02	Arachidonic acid metabolism
	1.365	500.2778	11.59	PE(20:4)	0.6	4.40E-02	Not known
	1.622	175.0251	1.94	Ascorbic acid	0.47	1.13E-02	Ascorbate and aldarate metabolism
	1.467	203.0822	3.17	Tryptophan	0.35	2.73E-02	Tryptophan metabolism
	1.421	131.0381	0.60	Glutaric acid	-0.33	3.41E-02	Fatty acid metabolism
	1.697	165.0405	14.40	Ribonic acid	-1.45	6.81E-03	Not konwn
**21I/21C(+)**	2.282	308.0909	0.92	Glutathione	1.84	8.35E-03	Cysteine and methionine metabolism
	2.019	258.1104	0.87	Glycerophosphocholine	1.35	2.83E-02	Not known
**21I/21C(-)**	1.631	214.0467	0.64	Glycerylphosphorylethanolamine	0.74	4.57E-02	Not known
	2.152	323.0298	0.70	Uridine monophosphate	0.73	2.01E-03	Not known
	1.751	243.0616	0.86	Uridine	0.71	2.76E-02	Pyrimidine metabolism
	1.964	149.0452	0.68	Ribose	0.4	8.80E-03	Carbon metabolism
	1.650	346.0557	0.72	dGMP	0.31	4.24E-02	Purine metabolism
	1.912	146.0459	0.67	Glutamate	0.24	1.21E-02	Alanine, aspartate and glutamate metabolism
	2.043	179.0552	0.64	α-D-Glucose	0.22	5.09E-03	Not known
	1.675	102.0559	0.67	γ-Aminobutryic acid	0.2	3.83E-02	Not known
	1.715	154.0618	0.61	Histidine	-0.21	3.23E-02	Histidine metabolism
	1.746	191.0198	0.81	Citric acid	-0.4	2.83E-02	TCA cycle
	1.663	500.2778	11.59	PE(20:4)	-0.44	4.02E-02	Not known
	2.017	128.0355	0.85	Pyroglutamic acid	-0.54	6.16E-03	Glutathione metabolism
	1.733	478.2933	12.08	PC(15:1)/ PE(18:1)	-0.62	3.00E-02	Not known
	1.654	452.2777	11.85	PC(13:0)/ PE(16:0)	-0.73	4.17E-02	Not known
	1.785	480.3088	12.71	PC(15:0)/ PE(18:0)	-0.78	2.36E-02	Not known
	1.774	218.103	2.18	Pantothenic Acid	-0.81	2.49E-02	beta-Alanine metabolism; Pantothenate and CoA biosynthesis
	1.641	436.2827	12.10	PE(P–16:0/0:0)	-0.89	4.38E-02	Not known
	1.904	476.2775	11.57	PE(18:2)	-1.19	1.27E-02	Not known

* indicates log2 fold change

+ indicates ESI+ mode

- indicates ESI- mode

On day 14 (R^2^X = 0.5, R^2^Y = 0.992, Q^2^ = 0.456 ESI+; R^2^X = 0.583, R^2^Y = 0.999,Q^2^ = 0.394 ESI- (Fig 3B), there were more altered metabolites. In the positive ion mode, levels of guanosine, guanine, hypoxanthine and inosine, important intermediates in purine metabolism, were shown to be more abundant in the infected mice group compared to those in the control group (Fig 4B). Other metabolites that are involved in crucial metabolic pathways, such as propionyl-L-carnitine, acetylcarnitine and eicosapentaenoic acid, had similar changes. PC (18:0), PE (21:0), PE (P–16:0), PC (15:0)/PE (18:0), PC (15:1)/PE (18:1) showed significantly increased levels. Meanwhile, the levels of some other kind of phospholipids were found to be lower in the infection group than the control group, including phosphatidylinositol (PI) (22:2), phosphatidic acid (PA) (18:3), PC (10:0) and PC (12:0). At the same time in the negative model, the levels of PE (P–16:0/0:0), docosahexaenoic acid, PC (13:0)/PE (16:0), PC (15:0)/PE (18:0) and PE (22:4) were found to be elevated. In contrast, the level of ribonic acid declined.

The number of differential metabolites in the infected mice brain decreased on day 21 (R^2^X = 0.921, R^2^Y = 1, Q^2^ = 0.73 ESI+; R^2^X = 0.55, R^2^Y = 0.988,Q^2^ = 0.572 ESI-) (Fig 3C). Under the ESI+ model, glutathione, which functions as an important antioxidant preventing damage to cellular components, was shown to be more abundant in the infection group than in the control group (Fig 4C). Another key metabolite, glycerophosphocholine, which plays an important role in improving the brain function, has the same profile. Under the ESI- model, levels of glycerylphosphorylethanolamine, uridine monophosphate and uridine were higher in infected mice (S2C Fig). The rare amino acid pyroglutamic acid (a metabolite in the glutathione cycle) was shown to be less abundant in the infection group. Metabolites that have the similar profiles included citric acid and pantothenic acid. Phospholipids that are a major component of all cell membranes were found to be down-regulated at this period, including PE (18:2), PE (P–16:0/0:0), PC (15:0)/ PE (18:0), PC (13:0)/ PE (16:0), PC (13:0), and PC (15:1) /PE (18:1).

### Differential expression of host brain metabolomes among *T*. *gondii* infected mice groups

**Fig 5:**
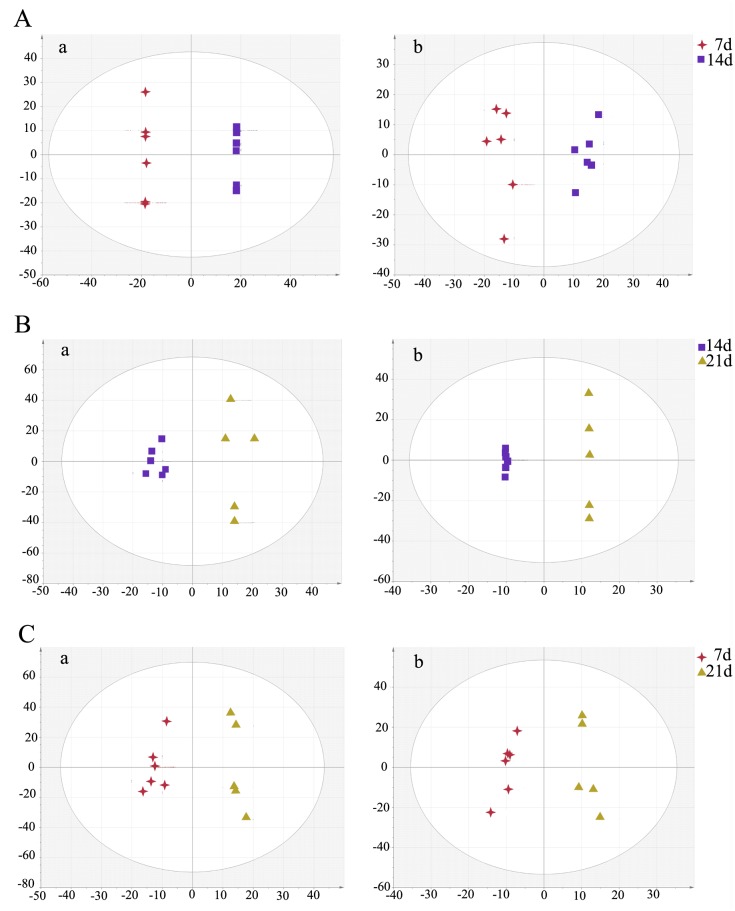
OPLS-DA score plot showed a clear separation between the infected brain samples at different time points. (A) The 7 day group and the 14 day group; (B) The 21 day group and the 14 day group; (C) The 21 day group and the 7 day group. In OPLS-DA score plot, each data point represents one mouse brain sample, and the distance between points in the plot indicates the similarity between samples. a. ESI+, b. ESI-. X axis is PC1, y axis is PC2.

**Fig 6:**
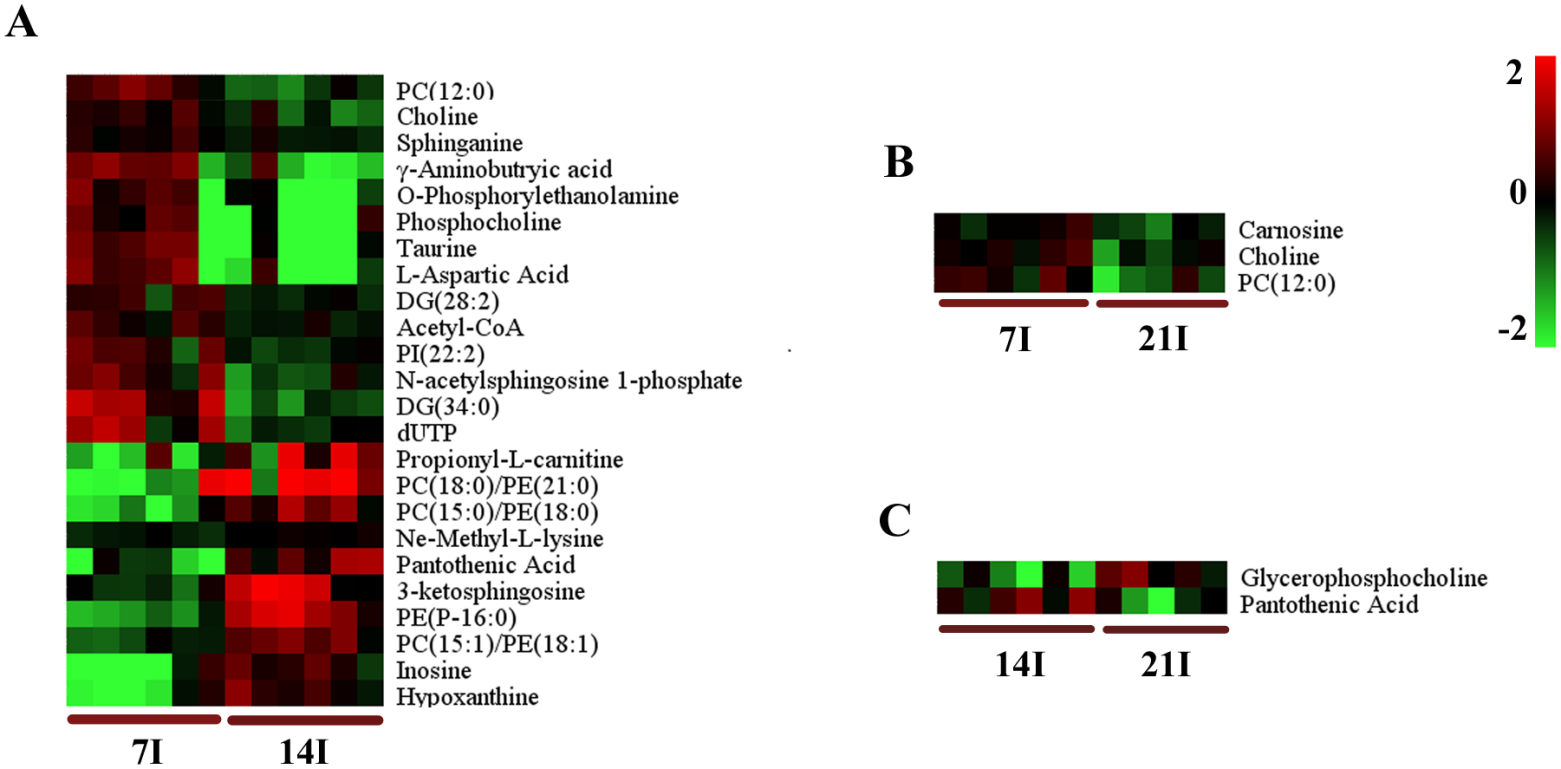
Heat map showing the significantly dysregulated metabolites identified between different infected groups in ESI+ mode. Heat maps show a clear separation of the metabolomic profile between different infected groups. Normalized metabolite abundance (log2 transformed and row adjustment) are visualized as a color spectrum and the scale from least abundant to highest ranges is from -2.0 to 2.0. Green color indicates low expression, and red color indicates high expression of the detected metabolites. (A) 7D infected group (7I) vs 14D infected group(14I); (B) 7D infected (7I) group vs 21D infected group (21I); (C) 14D infected group (14I) vs 21D infected group (21I).

To understand the metabolomic changes in the mice brain during different stages of *T*. *gondii* infection, a two-component OPLS-DA model was constructed to achieve a distinct separation between different infection groups (Fig 5). As shown in Table 2, there were many significant alterations in the brain levels of several metabolites, especially between 14 DPI group and 7 DPI group (R^2^X = 0.77, R^2^Y = 1, Q^2^ = 0.892 ESI+; R^2^X = 0.36, R^2^Y = 0.962,Q^2^ = 0.594 ESI-) (Fig 5A). In the positive ion mode, the levels of pantothenic Acid, propionyl-L-carnitine, 3-ketosphingosine, hypoxanthine and inosine were significantly higher in the brain on 14 DPI (Fig 6A). Up-regulated metabolites in negative ion mode included N-Acetylleucine, FMN and ascorbic acid. Meanwhile in the same ion mode, metabolites that were significantly down-regulated included taurine, L-Aspartic acid, ascorbic acid, phosphocholine, γ-aminobutryic acid, o-phosphorylethanolamine, dUTP, N-acetylsphingosine 1-phosphate, choline and acetyl-CoA. Most significantly altered phospholipids were up-regulated no matter in positive ion mode or negative ion mode (Fig 6A, S3A Fig). Only the levels of PC (12:0) and PI (22:2) (log2 fold change:-1.12, -0.74, respectively) were decreased. These results showed that phospholipids anabolism was active during the transitional phase from physical illness to recovery.

**Table 2:** List of identified metabolites that varied between different infection stages.

	VIP	m/z	Rt (min)	metabolites	F.C.*	p-value	Metabolic pathways
**14d/7d(+)**	1.344	524.3713	13.25	PC(18:0)/ PE(21:0)	2.36	3.00E-02	Not known
	1.857	438.2982	12.43	PE (P–16:0)	2.26	0.00E+00	Not known
	1.719	482.3246	13.09	PC(15:0)/ PE(18:0)	1.86	2.00E-03	Not known
	1.639	220.1185	2.32	Pantothenic Acid	1.78	4.00E-03	beta-Alanine metabolism; Pantothenate and CoA biosynthesis
	1.294	218.1388	0.95	Propionyl-L-carnitine	1.67	3.90E-02	Not known
	1.555	298.2745	10.47	3-ketosphingosine	1.65	8.00E-03	Not known
	1.536	137.0457	1.26	Hypoxanthine	1.38	9.00E-03	Purine metabolism
	1.509	269.0888	1.26	Inosine	1.34	1.10E-02	Purine metabolism
	1.815	480.3089	12.39	PC(15:1)/ PE(18:1)	1.12	1.00E-03	Not known
	1.791	161.128	0.62	Ne-Methyl-L-lysine	0.27	1.00E-03	Not known
	1.420	302.3059	10.64	Sphinganine	-0.34	2.00E-02	Sphingolipid metabolism
	1.497	810.1145	8.70	Acetyl-CoA	-0.5	1.20E-02	Taurine and hypotaurine metabolism; Glutathione metabolism
	1.415	509.4306	5.87	DG(28:2)	-0.53	2.00E-02	Not known
	1.507	104.1071	0.74	Choline	-0.69	1.10E-02	Glycine, serine and threonine metabolism
	1.440	653.3687	7.47	PI(22:2)	-0.74	1.80E-02	Not known
	1.544	422.2641	7.59	N-acetylsphingosine 1-phosphate	-1.04	9.00E-03	Not known
	1.752	454.2596	5.79	PC(12:0)	-1.12	1.00E-03	Not known
	1.533	468.992	5.57	dUTP	-1.28	1.00E-02	Pyrimidine metabolism
	1.335	142.0264	1.33	O-Phosphorylethanolamine	-1.38	3.20E-02	Glycerophospholipid metabolism
	1.534	104.0714	1.37	γ-Aminobutryic acid	-1.52	9.00E-03	Not known
	1.245	184.0735	1.37	Phosphocholine	-1.53	4.90E-02	Glycerophospholipid metabolism
	1.406	134.0449	1.38	L-Aspartic Acid	-1.73	2.20E-02	Alanine, aspartate and glutamate metabolism
	1.502	126.022	1.39	Taurine	-1.81	1.20E-02	Primary bile acid biosynthesis
	1.777	597.5586	7.76	DG(34:0)	-1.83	1.00E-03	Not known
**14d/7d(-)**	1.849	436.2827	12.10	PE(P–16:0/0:0)	3.71	6.74E-04	Not known
	1.682	172.0975	5.79	N-Acetylleucine	2.8	3.96E-03	Not known
	1.965	452.2777	11.85	PC(13:0)/ PE(16:0)	2.74	1.06E-04	Not known
	1.838	480.3088	12.71	PC(15:0)/ PE(18:0)	2.4	7.78E-04	Not known
	1.947	478.2933	12.08	PC(15:1)/ PE(18:1)	1.85	1.49E-04	Not known
	1.663	455.0968	4.85	FMN	1.69	4.67E-03	Riboflavin metabolism
	1.665	175.0251	1.94	Ascorbic acid	1.25	4.61E-03	Ascorbate and aldarate metabolism
**21d/14d(+)**	1.943	258.1104	0.87	Glycerophosphocholine	1.15	2.12E-02	Not known
	1.715	220.1185	2.32	Pantothenic Acid	-1.05	4.23E-02	beta-Alanine metabolism; Pantothenate and CoA biosynthesis
**21d/14d(-)**	1.727	214.0467	0.64	Glycerylphosphorylethanolamine	0.53	4.47E-02	Not known
	1.898	131.0380	0.60	Glutaric acid	0.38	2.28E-02	Fatty acid metabolism
	1.881	128.0355	0.85	Pyroglutamic acid	-0.43	2.46E-02	Glutathione metabolism
**21d/7d(+)**	1.159	227.1116	0.63	Carnosine	-0.46	3.20E-02	Histidine metabolism
	1.507	104.1071	0.74	Choline	-0.54	3.40E-02	Glycine, serine and threonine metabolism
	1.752	454.2596	5.79	PC(12:0)	-0.82	3.40E-02	Not known
**21d/7d(-)**	1.989	145.0143	0.83	Oxoglutaric acid	1.87	2.10E-02	TCA cycle
	1.843	214.0467	0.64	Glycerylphosphorylethanolamine	0.4	3.80E-02	Not known
	2.062	175.0251	1.94	Ascorbic acid	0.35	1.50E-02	Ascorbate and aldarate metabolism
	2.184	455.0968	4.85	FMN	0.29	8.00E-03	Riboflavin metabolism
	2.261	191.0198	0.81	Citric acid	-0.42	5.00E-03	TCA cycle
	1.805	739.4354	7.94	PA(40:10)	-1.12	4.30E-02	Not known

* indicates log2 fold change

+ indicates ESI+ mode

- indicates ESI- mode

Brain samples from 21 DPI group and 14 DPI group showed a very high segregation (R^2^X = 0.412, R^2^Y = 0.958, Q^2^ = 0.343 ESI+; R^2^X = 0.857, R^2^Y = 1, Q^2^ = 0.587 ESI-), as shown in Fig 5B. The same distinct metabolic profiles were not seen between 21DPI group and 7DPI group (R^2^X = 0.525, R^2^Y = 0.974, Q^2^ = 0.373 ESI+; R^2^X = 0.568, R^2^Y = 0.963,Q^2^ = 0.332 ESI-) (Fig 5C), in which Q^2^ were less than 0.4 in both ion modes. The number of significantly changed metabolites was very small between 21 DPI group and 14 DPI group. Glycerophosphocholine was the highest abundance metabolite (log2 fold change = 1.15) and pantothenic acid was the lowest (log2 fold change = -1.06) (Fig 6C). Comparisons between 21 DPI group and 7 DPI group showed that oxoglutaric acid, also called α-ketoglutarate (an intermediate in the Krebs cycle), was shown to be more abundant (log2 fold change = 1.87) (S3B Fig). The level of carnosine, choline and citric acid were slightly decreased. Glycerylphosphorylethanolamine, known to improve *in vivo* the learning and memory processes, was up-regulated.

### Identification of potential biomarkers

**Fig 7:**
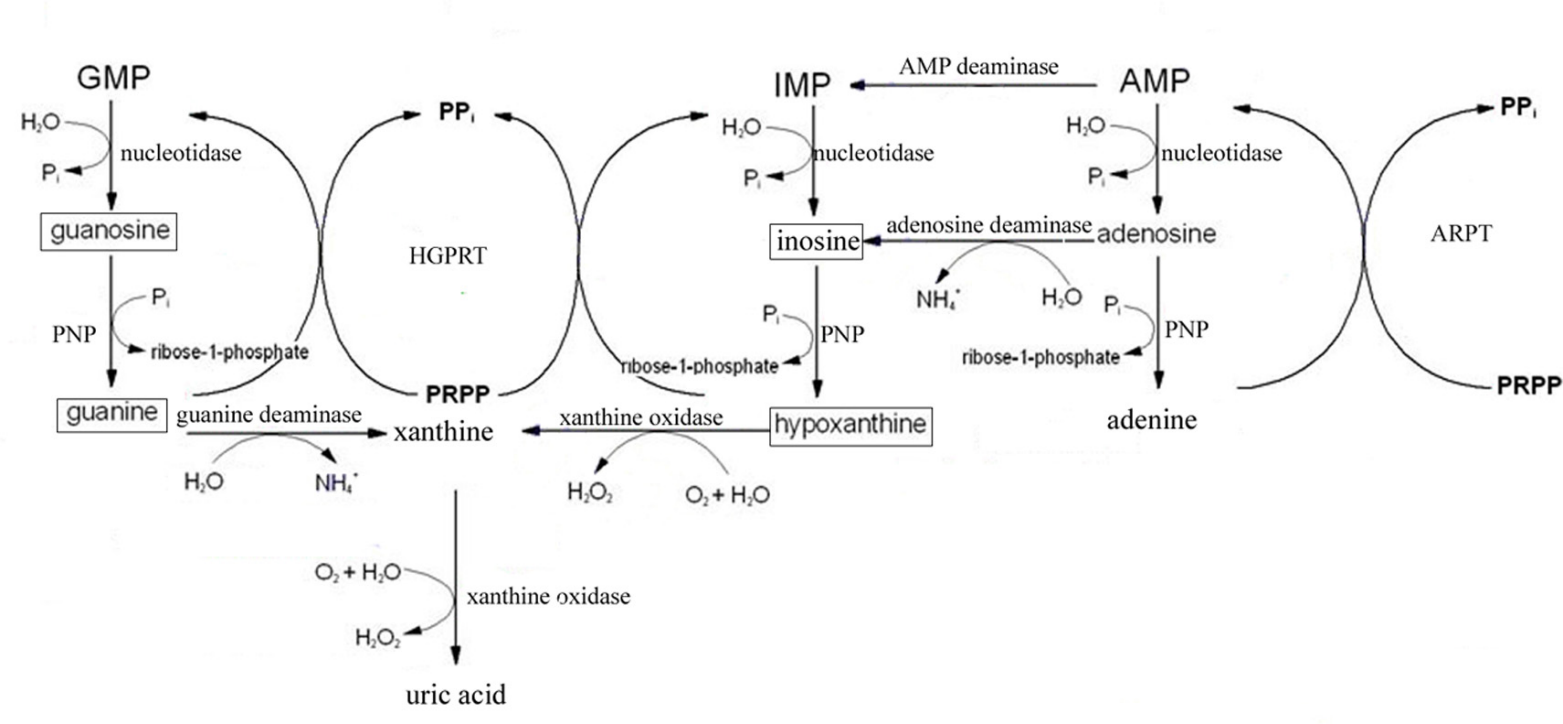
The schematic of purine metabolism diagram illustrating the biological significance of some biomarkers for prediction of physical recovery of illness. (□ means that the biomarkers were obtained in this experiment).

Infected mice began to restore their health on day 14, showing an increased appetite and enhanced mental state. We then investigated metabolites that have the potential to be used as biomarkers for the prediction of physical recovery from acute *T*. *gondii* infection. To select potential marker metabolites, differential metabolites identified on the 14 day were subjected to ROC analysis. The differentiation performance (specificity and sensitivity) was assessed by the area under the curve (AUC>0.85) and eight significantly differential metabolites were selected as potential biomarkers, as shown in Table 3. Some marker metabolites are involved in purine metabolism as illustrated in Fig 7.

**Table 3:** Marker metabolites found in LC/MS chromatograms of control and infected groups at 14DPI.

Ion mode	Metabolite	*R*t (min)	Chemical class	*P*-value^a^
**ESI(+)**	Propionyl-L-carnitine	0.952499	Fatty ester	0.028171
**ESI(+)**	Guanine	1.235958	Base	0.020226
**ESI(+)**	Guanosine	1.237643	Nucleoside	0.000632
**ESI(+)**	Inosine	1.261978	Alkaloid	0.000264
**ESI(+)**	Hypoxanthine	1.262799	Purine nucleoside	0.000646
**ESI(-)**	Ascorbic acid	1.937837	Vitamin	0.01134
**ESI(-)**	Docosahexaenoic acid	14.65844	Fatty acid	0.006129
**ESI(-)**	Arachidonic Acid	14.82713	Fatty acid	0.018173

^a^ Statistical *p*-value calculated using a two sample *t*-test (significance at *p* < 0.05).

## Discussion

In the present study, LC-MS-based metabolomic analysis technology was used to identify characteristics of the metabolite profiles of mice infected with *T*. *gondii* and to discover biomarkers that discriminate *T*. *gondii*-infected from non-infected controls. LC-MS based metabolomic profiling studies have become an increasingly powerful tool to analyze the interaction between parasites and their hosts, such as malaria caused by another apicomplexan protozoan *Plasmodium falciparum* [27,28] and human African trypanosomiasis (HAT) [29]. In these studies, marked alterations in host’s metabolic profiles were detected and some perturbed metabolites were identified as biomarkers with a potential diagnostic value. Likewise, in our study we have profiled the dynamic metabolic changes in mice brain infected by *T*. *gondii* Pru strain over the course of 21 DPI. We found metabolic shifts between *T*. *gondii* infected and non-infected mice as well as between *T*. *gondii* infected mice at different time points post infection.

Infected mice began to exhibit clinical signs of toxoplasmosis at 7 DPI. As the disease progresses, mice exhibited typical symptoms of acute infection around 11 DPI. Although some mice also become more dispirited, less energetic, and exhibited weight loss and reduced physical activity at 14 DPI, they appeared to regain health. Most infected mice regain health at 21 DPI, which correlated with increased appetite and improved physical activity. Pathological studies demonstrated significant structural changes occurred by 21 DPI, including inflammatory cell infiltration and the presence of parasite cysts, which are correlated with the changes of brain metabolism.

Brain samples were collected at different time points (7, 14 and 21 DPI) and analyzed to profile the parasite-induced metabolite changes related to the brain injury. OPLS-DA as a supervised pattern recognition process was necessary to maximize the variation associated with the mice health status. Each data point represents one brain sample, and the distance between points in the score plot is an indication of the similarity between samples. The score plots showed that all the infected groups could be easily distinguished from the corresponding control groups and the infected groups at different time points could also be clearly separated.

Both *T*. *gondii* infected and uninfected mice brains were analyzed to determine the parasite-induced alterations to normal brain metabolism. Mice at the early infection stage (7 DPI) showed a slight shift in metabolism, which was marked by an increase of UDP-N-acetyl-D-galactosamine (ESI-) and a decrease of leukotriene D4 (ESI+). Most of the detected metabolites showed peak or minimum abundance at 14 DPI, a period close to the acute infection [30]. The metabolome closely reflects the activities of the brain at the functional level and the metabolites that fluctuated with disease progression are indicative of the major metabolic processes that are closely related to the regulation of *T*. *gondii* infection. For example, cytidine, a decomposition product of cytidine monophosphate (CMP), was down-regulated along with the L-aspartic acid, which is an important building block of the *de novo* pyrimidine biosynthetic pathway in *T*. *gondii*. Arginine follows a similar profile. Previous studies showed arginine starvation could trigger differentiation of tachyzoites to bradyzoites, the slowly replicating stage contained within stable cyst-like structures [31]. These findings demonstrate that alteration of host metabolites (e.g. arginine) can modulate *T*. *gondii* phenotype (tachyzoite-bradyzoite) switching, an integral part of the infection process, which enables the parasite to hide inside host cells.

It is well established that the glucose is the primary energy source for brain. However, under starvation or fasting conditions, fatty acids could substitute glucose as an alternative substrate for brain energy metabolism. With regard to dietary intake by infected mice and energy demands for survival, the metabolite profiles of carnitine and lipid species in the brain is very worthy of attention. Significantly altered carnitines and phospholipids indicate variation of fatty acids oxidation metabolism and phospholipids metabolism, respectively. For example, acetyl-carnitine, an acetyl derivative of L-carnitine, was found to be more abundant in the infected mice group, which is consistent with others [32]. Acetyl-carnitine is involved in fatty acids oxidation metabolism, in which its acetyl groups are mostly incorporated into saturated fatty acid [33]. Apart from its role in reducing oxidation of glucose, acetyl-carnitine also takes part in many neuro-protective roles [34–36]. Propionyl-L-carnitine, playing a significant role in fatty acid oxidation and energy expenditure, were also shown to be more abundant at the same time [37]. Phospholipid is a major component of all cell membranes as it forms the phospholipid bilayer. Variations of PC, PI, PA and PE levels were observed between infection mice and the healthy ones (Table 1). The precursors of membrane phospholipids, phosphocholine and phosphoethanolamine, were down-regulated due to the presence of *T*. *gondii* infection. Phosphocholine is an intermediate in the synthesis of phosphatidylcholine, during which ATP and choline are converted into ADP and phosphocholine via catalysis by choline kinase. Phosphatidylethanolamine, synthesized by phosphoethanolamine, is found in all living cells, especially in nervous tissues. The decline of these two metabolites might be bound up with reduced food intake.

Mentally, infected mice exhibited lethargy and energetic dispirited, which might link with the metabolites variations. For instance, taurine is the second most abundant amino acid in the CNS, which showed significantly decreased levels at this period. Taurine functions as a key regulator of neuronal activity, neuromodulator and osmoregulator [38]. Moreover, it takes part in the development and survival of neural cells [39]. Gama-Aminobutyric acid (GABA), as the main inhibitory neurotransmitter in the CNS, also showed similar profile, which is found to function in reducing neuronal excitability [40].

The physical condition of infected mice becomes improved by 21DPI. The number of metabolites with statistically significant variations in abundance decreased at this period. Metabolites that were involved in the restoration of physical fitness of mice were elevated. For example, glutathione, as an important antioxidant, was up-regulated in the infected mice. Glutathione assists in the defense against reactive oxygen species generated during oxidative metabolism and also plays an important role in DNA repair and protein synthesis [41–43].

Direct comparisons of metabolite levels between day 14 and day 7 PI showed the most dramatically changed metabolites as shown in Table 2. Pantothenic acid, also known as vitamin B5, was significantly elevated with the log2 fold change of 1.78. Pantothenic acid is required for the synthesis of coenzyme A (CoA) that is involved in metabolism of fats, carbohydrates, and amino acids [44]. Acetyl coenzyme A (Acetyl-CoA) derived from CoA under acylation reaction was down-regulated. This metabolite participates in many essential metabolic pathways, such as citric acid cycle, glycolysis and fatty acid metabolism. Acetyl-CoA is also involved in the biosynthesis of the neurotransmitter acetylcholine. Choline and phosphocholine are other two metabolites that showed lower abundance, which are involved in the synthesis of acetylcholine. The deficiency of these metabolites might be directly bound up with abnormal physical activities. Oxoglutaric acid, also called α-ketoglutaric acid was the highest abundance metabolite when comparison was made between day 21 and day 7 PI. Oxoglutaric acid is an important intermediate in the tricarboxylic acid cycle and is involved in the synthesis of glutamine. The number of significantly changed metabolites between 21 and 14 DPI is small, which might account for health recovery at the metabolism level.

On day 14 PI, although demonstrating obvious pathogenic signs, more than half infected mice had already begun to recover. Marker metabolites that were responsible for the separation of the day 14 infected mice group from the control group were summarized in Table 3. The levels of all these metabolites were found to be statistically different (*p* < 0.05) and marker metabolites selected in this period were involved in many crucial metabolic pathways. For example, guanosine, guanine, hypoxanthine and inosine, intermediates in the purine salvage pathway, reached their peak abundance at 14 DPI. For the host, purine salvage pathway is the main way for purine synthesis in the brain. Purine salvage pathway is considered more energy efficient and integral to the cause or treatment of a number of human diseases of purine metabolism [45]. Because *T*. *gondii* is not able to synthesize purines *de novo*, it is more reliant on the purine metabolic enzymes and intermediates in the purine salvage pathway to ensure growth [46]. As the parasite’s sole source of purine bases, this salvage pathway is also essential for cyst growth and has been considered a potential target of new anti *T*. *gondii* drugs [47].

In conclusion, metabolomic analysis yielded a clear separation between non-infected and *T*. *gondii*-infected mice groups, indicating a substantial imprint from the infection on the metabolism of mice during the progression of the disease. In a longitudinal study, the strongest separation occurred around 14 DPI. Altered metabolomic pathways included changes in carbohydrate metabolism, lipid metabolism, energetic metabolism and fatty acid oxidation. Perturbed metabolites and pathways detected in this study may provide valuable information for discovery of new targets for novel therapeutic and diagnostic applications. Although biomarker discovery using experimental infections has the benefit of documenting when the biomarker detects the infection, validation of the use of metabolomics to detect infection under field conditions will need to be done to ultimately demonstrate its utility.

## Supporting Information

S1 Fig Differential metabolic profiles in mice brain among the six mice groups and during the time courses of toxoplasmosis. (A) LC-MS based PCA scores of brain of mice with/without infection with *T*. *gondii* in positive ion mode. (B) LC-MS based PCA scores of brain of mice with/without infection with *T*. *gondii* in negative ion mode. QC means quality control samples.
(TIF)

S2 Fig Comparison of the mice brain metabolomes during the course of infection. Heat maps representing the significantly changed metabolites between infected groups and the corresponding control groups in ESI- mode (A, B and C). Normalized metabolite abundance (log2 transformed and row adjustment) are visualized as a color spectrum and the scale from least abundant to highest ranges is from -2.0 to 2.0. Green indicates low expression, whereas red indicates high expression of the detected metabolites. (A) 7D infected group (7I) vs 7D control (7C); (B) 14D infected group (14I) vs 14D control (14C); (C) 21D infected group(21I) vs 21D control (21C).
(TIF)

S3 Fig Heat map showing the significantly dysregulated metabolites identified between different infected groups in ESI- mode. Heat maps show a clear separation of the metabolomic profile between different infected groups. Normalized metabolite abundance (log2 transformed and row adjustment) are visualized as a color spectrum and the scale from least abundant to highest ranges is from -2.0 to 2.0. Green color indicates low expression, and red color indicates high expression of the detected metabolites. (A) 7D infected group (7I) vs 14D infected group(14I); (B) 7D infected (7I) group vs 21D infected group (21I); (C) 14D infected group (14I) vs 21D infected group (21I).
(TIF)
