# Supplementary figures and images for: Global Metabolomic Profiling of Mice Brains following Experimental Infection with the Cyst-Forming Toxoplasma gondii
Source: PLoS One. 2015 Oct 2;10(10):e0139635. doi: 10.1371/journal.pone.0139635 (PMC4592003; doi:10.1371/journal.pone.0139635)

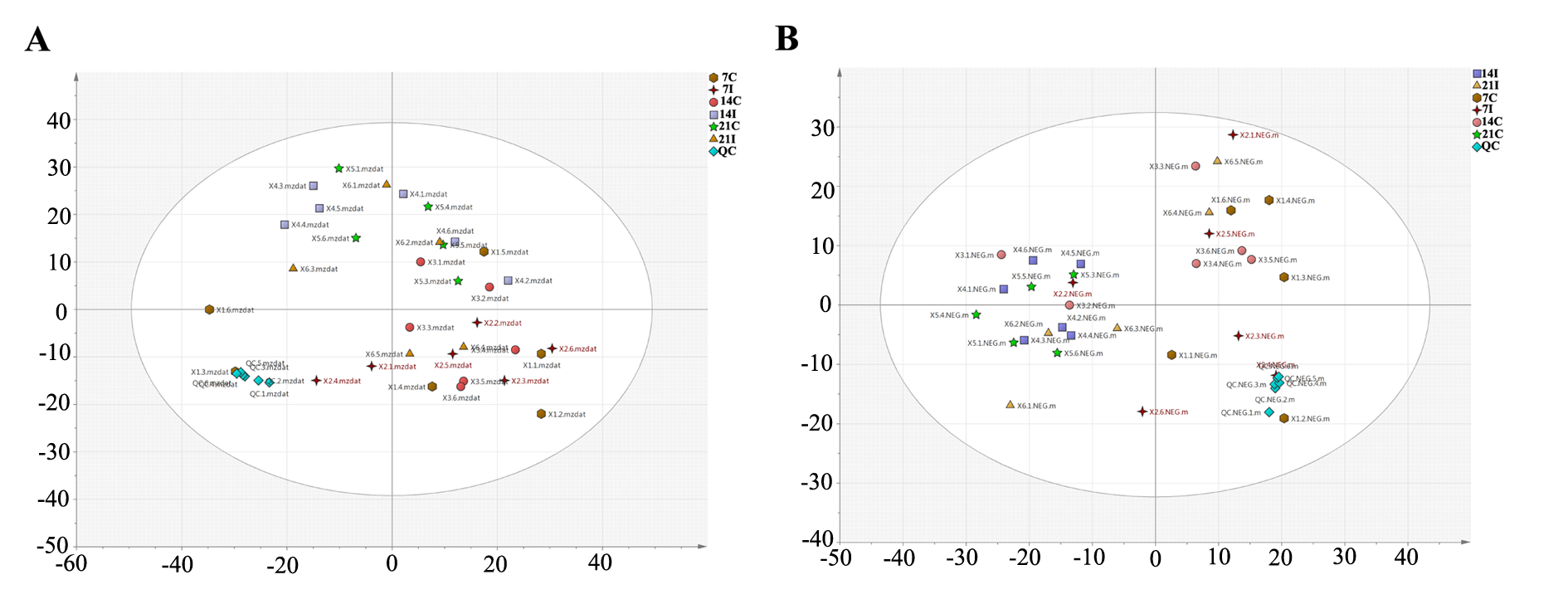

Supplement: S1 Fig — (A) LC-MS based PCA scores of brain of mice with/without infection with T. gondii in positive ion mode. (B) LC-MS based PCA scores of brain of mice with/without infection with T. gondii in negative ion mode. QC means quality control samples. (TIF) [file pone.0139635.s001.tif]

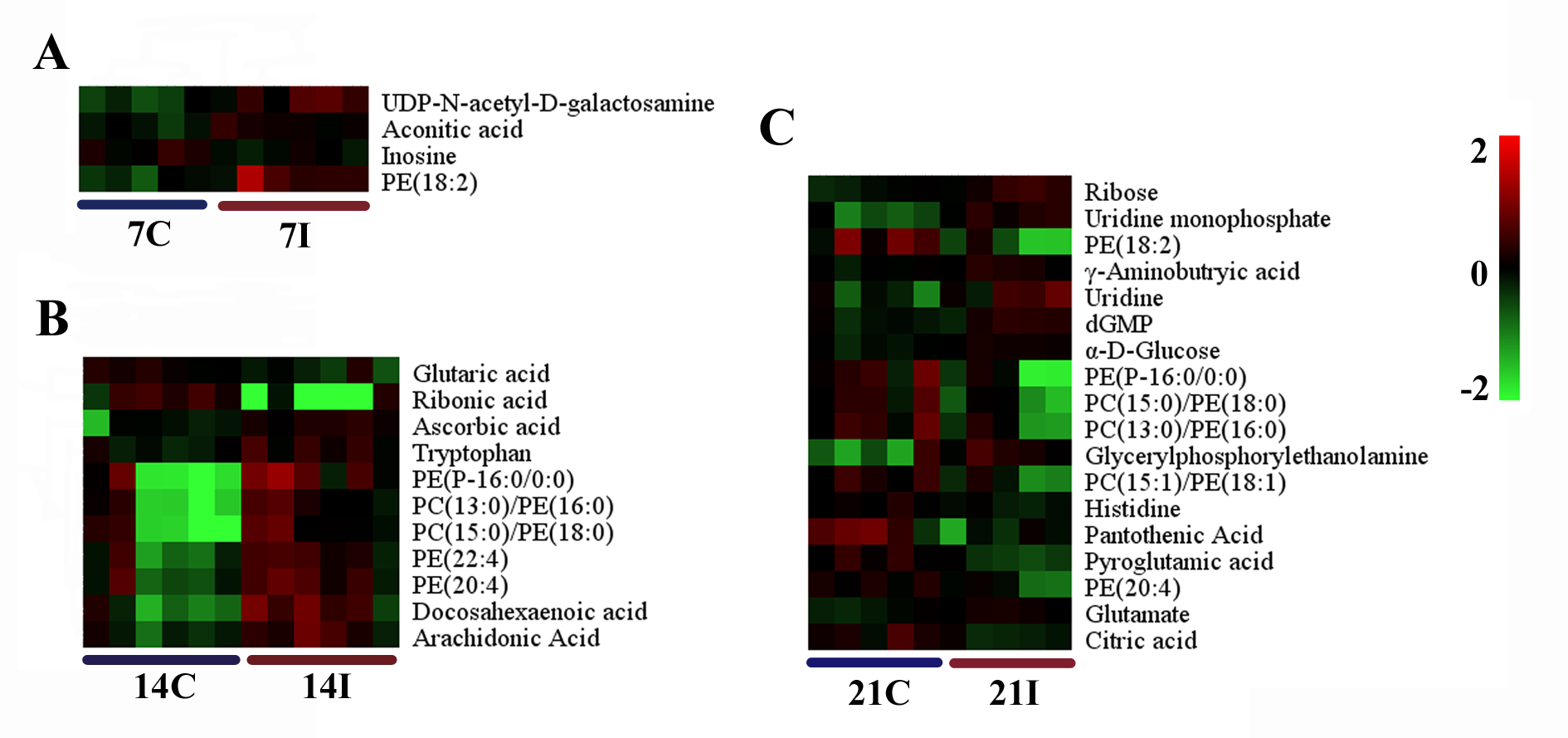

Supplement: S2 Fig — Heat maps representing the significantly changed metabolites between infected groups and the corresponding control groups in ESI- mode (A, B and C). Normalized metabolite abundance (log2 transformed and row adjustment) are visualized as a color spectrum and the scale from least abundant to highest ranges is from -2.0 to 2.0. Green indicates low expression, whereas red indicates high expression of the detected metabolites. (A) 7D infected group (7I) vs 7D control (7C); (B) 14D infected group (14I) vs 14D control (14C); (C) 21D infected group(21I) vs 21D control (21C). (TIF) [file pone.0139635.s002.tif]

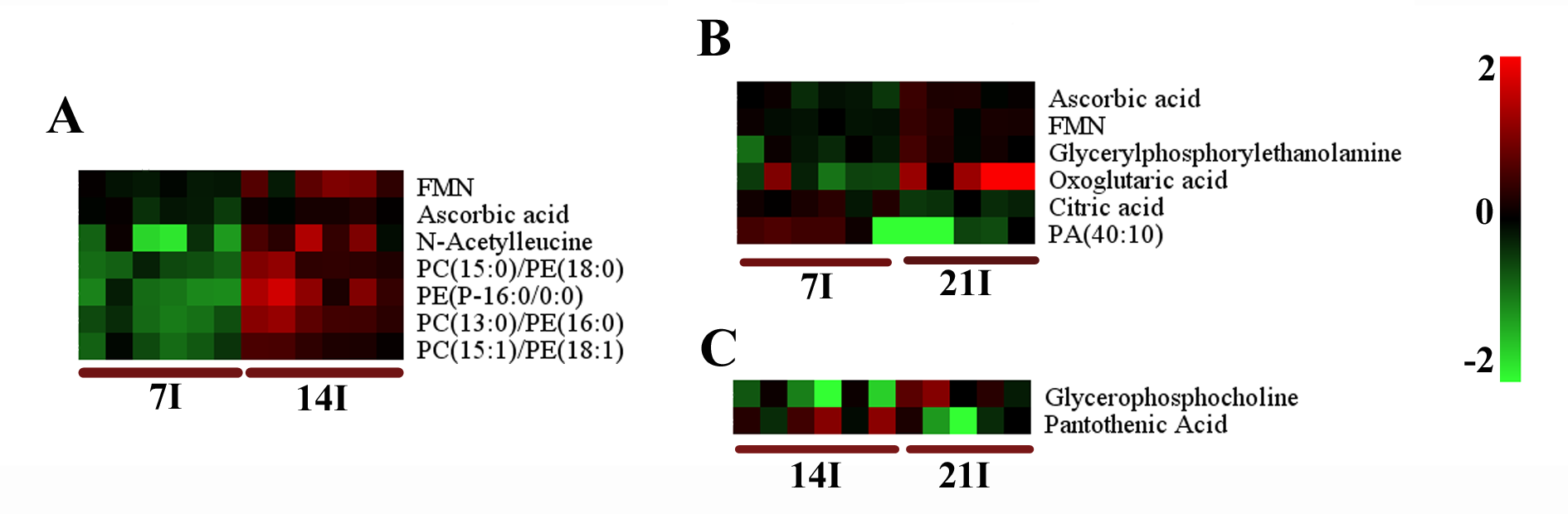

Supplement: S3 Fig — Heat maps show a clear separation of the metabolomic profile between different infected groups. Normalized metabolite abundance (log2 transformed and row adjustment) are visualized as a color spectrum and the scale from least abundant to highest ranges is from -2.0 to 2.0. Green color indicates low expression, and red color indicates high expression of the detected metabolites. (A) 7D infected group (7I) vs 14D infected group(14I); (B) 7D infected (7I) group vs 21D infected group (21I); (C) 14D infected group (14I) vs 21D infected group (21I). (TIF) [file pone.0139635.s003.tif]
